# Species-rich bark and ambrosia beetle fauna (Coleoptera, Curculionidae, Scolytinae) of the Ecuadorian Amazonian Forest Canopy

**DOI:** 10.3897/zookeys.1044.57849

**Published:** 2021-06-16

**Authors:** Stephanie A. Dole, Jiri Hulcr, Anthony I. Cognato

**Affiliations:** 1 Department of Entomology, California Academy of Sciences 55 Music Concourse Drive, San Francisco, CA 94118, USA Department of Entomology, California Academy of Sciences San Francisco United States of America; 2 School of Forest Research and Conservation, University of Florida 136 Newins-Ziegler Hall, Gainesville, FL 32611, USA University of Florida Gainesville United States of America; 3 Department of Entomology, Michigan State University 288 Farm Ln. East Lansing, MI 48824, USA Michigan State University East Lansing United States of America

**Keywords:** ambrosia beetles, bark beetles, diversity, neotropical, rainforest, Terry Erwin

## Abstract

Canopy fogging was used to sample the diversity of bark and ambrosia beetles (Coleoptera, Curculionidae, Scolytinae) at two western Amazonian rainforest sites in Ecuador. Sampling was conducted by Dr Terry Erwin and assistants from 1994–2006 and yielded 1158 samples containing 2500 scolytine specimens representing more than 400 morphospecies. Here, we analyze a subset of these data representing two ecological groups: true bark beetles (52 morphospecies) and ambrosia beetles (69 morphospecies). A high percentage of these taxa occurred as singletons and doubletons and their species accumulation curves did not reach an asymptote. Diversity estimates placed the total scolytine species richness for this taxon subset present at the two sites between 260 and 323 species. The α-diversity was remarkably high at each site, while the apparently high β-diversity was an artifact of undersampling, as shown by a Monte Carlo resampling analysis. This study demonstrates the utility of canopy fogging for the discovery of new scolytine taxa and for approximate diversity assessment, but a substantially greater sampling effort would be needed for conclusive alpha as well as beta diversity estimates.

## Introduction

First pioneered by Southwood (1961) in temperate forests and then later adapted for tropical research by numerous others (Lowman and Wittman 1996), canopy fogging methods use insecticide to collect arthropods from the upper architecture of the forest habitat. These methods have been used in surveying arthropod diversity, particularly in lowland rain forests and at several neotropical localities (Erwin 1982; Basset 2001; Rice 2015). These data in part were used to make Erwin’s (1982) bold estimate of 30–50 million arthropod species which was a stark contrast to previous estimates of 1.5–10 million (Erwin 1982). Although this provocative publication produced immediate criticism, it initiated focused scientific inquiry for an improved estimate of the number of worldwide arthropod species (e.g., May 1988; Adis 1990; Gaston 1991; Erwin 1991; Ødegaard 2000; Hamilton et al. 2010).

For a more than a decade, Erwin and colleagues sampled arthropod diversity in the Ecuadorian Amazon rainforest canopy at two lowland sites with identified trees, separated by 21 km of contiguous primary forest, using a standardized insecticidal fogging protocol (Erwin 1983a, 1983b; Pitman et al. 2001; Erwin et al. 2005). Sampling occurred during dry, wet, and transitional seasons to measure temporal turnover in species composition. Over nine million specimens were collected from these fogging events (Rice 2015) and many new taxa have been described from the specimens (e.g., Erwin 2010).

The Scolytinae (Coleoptera, Curculionidae) is comprised of approximately 257 genera containing 6000 species worldwide (Hulcr et al. 2015). Scolytine species feed sub-cortically on a wide variety of woody and herbaceous plants. The typical life cycle of scolytines consists of a brief dispersal flight period after adult emergence, followed by colonization of new hosts. Once a suitable host has been located, adults bore galleries into the host tissue, where eggs are laid, and larvae complete their development into the next generation of adults (Kirkendall et al. 2015).

Scolytines are divided into two main ecological groups: bark and ambrosia beetles. The degree to which scolytines specialize on specific hosts varies considerably depending on these ecological groups. Bark beetles bore into the phloem of trees, feed on tree tissues, and tend to have more specialized host preferences. Ambrosia beetles bore into the xylem, feed on species of symbiotic fungi, which grow along the walls of their galleries, and tend to have more generalized tree host preferences (Beaver 1979; Hulcr et al. 2007; Kirkendall et al. 2015). The majority of scolytine diversity occurs in tropical regions of the world, and many tropical species remain undescribed (Wood 2007; Hulcr et al. 2015; Smith et al. 2017). The Ecuadorian canopy fogging samples collected in Erwin’s surveys offer a rich source of new scolytine specimens because of observed scolytine species specialization to tree taxa, tree parts, and other microclimate factors (e.g., Hulcr et al. 2007; Kirkendall et al. 2015). In addition, only fewer than 200 species have been recorded for Ecuador (Wood 2007; Martínez et al. 2019; Jordal and Smith 2020), but the Ecuadorian species richness is likely underestimated given the continuous discovery of new species (e.g., Petrov and Flechtmann 2013; Petrov and Mandelshtam 2018; Smith and Cognato, in prep.).

Remarkably few studies have addressed the spatial and temporal turnover of scolytine species (β-diversity) (Deyrup and Atkinson 1987; Thunes 1998; Peltonen et al. 1998; Hulcr et al. 2008a, b). The majority of studies have focused on the composition of the scolytine fauna of temperate regions and examined the distributions of a few economically important species (Deyrup and Atkinson 1987; Jakus 1998; Peltonen et al. 1998; Oliver and Mannion 2001; Gaylord et al. 2006). Several studies in the tropics have attempted to determine the effects of seasonal changes in rainfall and temperature on the composition of scolytine communities (Beaver and Löyttyniemi 1991; Madoffe and Bakke 1995; Morales et al. 2000; Flechtmann et al. 2001; Hulcr et al. 2008a; Sandoval Rodríguez et al. 2017; Martinez et al. 2019; Sanguansub et al. 2020). Studies in Malaysian forests have examined the spatial distribution of scolytines across horizontal (Maeto et al. 1999; Chung 2004) and vertical gradients (Maeto and Fukuyama 2003; Simon et al. 2003). Recent studies in Thailand (Hulcr et al. 2008a; Sanguansub et al. 2020) and Papua New Guinea (Hulcr et al. 2008b) have used quantitative sampling to examine scolytine community composition and attempt to determine the proximate causes of the distribution of species in tropical habitats. Scolytine communities in lowland homogeneous Papua New Guinean rainforests have a low β-diversity, thus contradicting the expected trend of high β-diversity in the tropics. Novotny et al. (2007) similarly demonstrated low species turnover for insect communities when examined on a large scale across Papua New Guinea. In Thailand, two study sites separated by only 5 km were found to have significantly different scolytine species composition (Hulcr et al. 2008a), however the differences were attributed to differences in forest type, elevation, seasonality, mean annual temperature and humidity (Hulcr et al. 2008a; Sanguansub et al. 2020). Given the scenarios above, it is uncertain whether Amazonian forests will produce the same trend of low β-diversity seen in Papua New Guinea or high β-diversity, as was observed between different Thai forests.

In tropical ecosystems, an increase in host specificity combined with an increase in plant diversity is often used to explain high levels of species diversity. However, bark and ambrosia beetles show a reverse trend with at least some groups exhibiting lower host specificity in tropical regions. This is largely due to the greater abundance of ambrosial feeding scolytines which, as discussed above, tend to be relative host plant generalists (Beaver 1979), with high symbiont specificity. Nevertheless, most scolytine groups have higher species diversity in tropical regions than they do in temperate ones (Beaver 1979; Deyrup and Atkinson 1987; Hulcr et al. 2015). It is therefore expected that canopy fogging will yield a high number of scolytine species, but that the β-diversity of scolytines across the Amazonian canopy may not necessarily be high.

In this study, we use a subset of scolytine specimens representing both the bark beetle and ambrosia beetle ecological groups to assess the diversity of scolytines at two western Amazonian forest study sites. We use these data to assess the value of canopy fogging as a source of scolytine specimens, determine the spatial turnover of scolytine species, and use the data collected from these fogging stations to predict the scolytine species richness at the two sites.

## Materials and methods

### Field sites and sampling

The two study sites in this investigation were typical lowland rain forest habitats in the western Amazon Basin at the margin of Yasuní National Park, separated by 21 km of contiguous primary forest: Onkone Gare Station (cited as “Piraña” in Pitman et al. 2001 and Erwin et al. 2005) (0°39'25.685"S, 76°217'10.813"W; 216 m) and Tiputini Biodiversity Station (0°37'55.397"S, 76°08'39.205"W; 216 m). The Onkone Gare and Tiputini study plots were established in 1994 and 1997, respectively. The study sites receive an average of 2.7 m of rainfall per year (Erwin et al. 2005). Precipitation at the two sites is seasonal, with the wet season occurring from May to October and the dry season occurring from November to April.

Tree data were recorded for collecting stations within Erwin’s canopy fogging study transects. Trees with a diameter at breast height (diameter measured at 1.33 m from tree base) greater than 10 cm that had at least part of a branch hanging over the collecting sheet were tagged by Erwin’s team and subsequently identified (Pitman et al. 2001). In terms of trees, the Onkone Gare and Tiputini sites are very different at the species level (73% occurring at only one site), moderately dissimilar at the generic level (53% occurring at only one site), and fairly similar at the family level (26% occurring at only one site). With approximately 250 species each, the two study sites represent individually 21.26% (Onkone Gare) and 21.42% (Tiputini) and collectively 34% of the regional tree diversity (Erwin et al. 2005).

The fogging protocol followed Erwin (1983a, b). The study plot area at each site (100 m × 1000 m) was divided into 10 transects (10 m × 100 m). Each transect consisted of 10 collecting stations, catches for falling arthropods (3 m × 3 m), which were randomly arrayed on both sides of the transect centerline. Each station was constructed of a sampling sheet tied, suspended 1 m off the ground, and fixed with a collecting jar attached to the center of the sheet. The total area of these collecting stations was 9250 m^2^, which represented just 1.11% of the entire transect at each site.

Fogging occurred at Onkone Gare from 1994–1996 and 2005–2006, and at Tiputini from 1998–2002 three times per year: January/February (dry), June/July (wet), and October (transitional). Foggings occurred at 0345–0500 hr in order to minimize insecticidal drift outside of the column due to air currents. The pyrethroid insecticide resmythrim was fogged for 60 seconds in a column from just above the sheet to a height that was then recorded for each fogging event (for details on fogging techniques and equipment used see Lucky et al. 2002). Previous studies demonstrated that arthropod repopulation occurs within 10 days after fogging (Lucky et al. 2002). Hence, the same stations could be resampled seasonally without an effect on sampling.

The samples examined herein represent only a subset (1158 samples) of the total (1400+ samples) taken during the decade of canopy fogging. Samples from several collecting expeditions were not exported from Ecuador and were therefore not available for this study. Scolytine beetles were extracted from the adult Coleoptera samples, sorted to morphospecies, and identified using published keys (e.g., Wood 2007). For this study, subset of scolytine specimens representing both the bark beetle (Bothrosternini, Phloeosinini, Phloeotribini, Phrixosomini) and ambrosia beetle (Xyleborini, Ipini: *Premnobius*) were included for the diversity assessments. We excluded the Corthylini, Trypophloeini, Hexacolini and other former Cryphalini, and Scolytini because of potential inflation of the diversity estimates due to sexual dimorphism and cryptic species boundaries.

### Analysis of communities

We compared the differences between the communities of scolytines occurring at Onkone Gare and Tiputini with statistical analyses used in similar studies (Hulcr et al. 2008a, b). All calculations were performed with the EstimateS software (Colwell 2004).

Accumulation curves of species richness were estimated using the Mao Tau function which is an analytical analog of a randomized rarefaction procedure. Impact of rare species on these accumulation curves was evaluated with abundance-based, Chao1 (Chao 1984) and the Abundance-based Coverage Estimator (ACE) (Chao and Lee 1992; Chao et al. 1993), and incidence-based, Chao2 (Chao 1987) and the Incidence-based Coverage Estimator (ICE) (Lee and Chao 1994), richness estimators. ACE and ICE considered two classes of species for their calculations: those that are rare and those that are not rare. For analytical purposes, ACE and ICE considered species with fewer than 10 individuals in the sample to be rare (the default setting in EstimateS Colwell 2004). Chao1 and Chao2 treat all species (rare and not rare) the same in their calculations. These calculations also estimate the effect of unseen rare species (rare species that were not sampled). The default bias-corrected option was used to calculate Chao1 and Chao2. This analysis estimated the critical value for the abundance distribution as > 0.5 for all subsets of the data analyzed. In cases such as these the recalculation of the Chao1, Chao2, ACE, and ICE using the Classic option is recommended. Thus, the richness estimators reported herein were calculated using the Classic option under diversity settings in EstimateS (Colwell 2004).

In addition to the above richness estimators, a second-order Jackknife (Burnham and Overton 1978, 1979) was calculated. This measure is based on the number of species that occur in one sample, as well as those that occur in exactly two samples. Colwell and Coddington (1994) have shown the second-order Jackknife to be one of the more reliable predictors of species diversity.

Faunal distinctness or dissimilarity between the two sites was measured with the Complementarity Index (CI) (Colwell and Coddington 1994). This calculation is based on the observed species for each sample, the total species richness, and the number of species unique to either sample. Complementarity Index value of 1 indicates that compared samples do not share any species in common. Complementary indices were calculated for each taxon, as well as for the ambrosia beetles, bark beetles, and for the total sample (all beetles) for Onkone Gare and Tiputini. In addition, for the total sample, we used the Chao-Sørensen abundance-based estimator which corrects for biases of incomplete samples of the fauna by incorporating the effect of unseen shared species (Chao et al. 2005, 2006). Given that the Onkone Gare study site was sampled more than twice as much as the Tiputini site (311 lots versus 115 lots, respective), we rarefied the data for the Chao-Sørensen analysis. A lognormal distribution was used for rarefaction resulting in a Tiputini mean (SD) = 1.93 (12.21) and a Onkone Gare mean (SD) = 1.93 (12.70)]. The total number of individual specimens for both the Tiputini and the Onkone Gare rarefied samples equaled 230.

To test the hypothesis that the observed similarity is not statistically significant, but rather a result of random sampling of individuals from similar or identical scolytine communities, we performed a Monte Carlo analysis using the total rarefied dataset. In each replicate of this test individuals of each species were randomly distributed between the two sites. This randomized dataset was then used to calculate the Chao-Sørensen similarity index and the procedure was repeated 100 times.

## Results

A total of 1158 canopy fogging bulk samples were analyzed from the Ecuadorian Amazon study transects; 965 from Onkone Gare and 293 from the Tiputini (Table 1). Scolytines were found in 60% of the Onkone Gare samples, 75% of the Tiputini samples, and 69% of the total samples from both sites. These samples contained a total of 2500 scolytines, representing more than 400 morphospecies including a large number of rare species occurring as singletons and doubletons. The subset of taxa examined here represented 688 individuals and 121 species (Tables 2, 3). The bark beetles (Bothrosternini, Phloeosinini, Phloeotribini, and Phrixosomini) totaled 183 specimens representing 9 genera and 52 morphospecies (Tables 2, 4). The ambrosia beetles (Xyleborini, and Premnobiina) totaled 504 specimens representing nine genera and 70 species (Tables 3, 4).

**Table 1.** Numbers of scolytine species from canopy fogging two sites in Ecuador, complimentary index values and species richnessestimates. CI= complimentary index, ACE= Abundance-based Coverage Estimator, ICE= Incidence-based Coverage Estimator, Jack2 = second-order jackknife.

**(A) All taxa**	**No. Samples**	**Species Observed**	**Unique Species**	**CI**	**ACE**	**ICE**	**Chao1**	**Chao2**	**Jack2**
Onkone Gare	965	98	74	NA	275	292	296	309	217
Tiputini	293	47	24	NA	90	96	85	90	95
Both Sites	1158	121	NA	0.81	301	323	308	311	260
**(B) Bark beetles**									
Onkone Gare	965	42	32	NA	95	90	154	109	83
Tiputini	293	20	10	NA	38	35	32	30	35
Both Sites	1158	52	NA	0.81	121	119	154	137	109
**(C) Ambrosia beetles**									
Onkone Gare	965	56	42	NA	179	184	153	208	128
Tiputini	293	27	14	NA	51	61	55	67	58
Both Sites	1158	69	NA	0.81	183	211	161	174	150

**Table 2.** Numbers of bark beetle species collected at two sites in Ecuador.

**Subtribe**	**Genus**	**Species**	**Locality(ies)**	**No. Specimens**
Bothrosternini	* Akrobothrus *	*ecuadoriensis*	Onkone Gare	3
	* Bothrosternus *	n. sp. nr. truncatus	Onkone Gare/Tiputini	8
		sp. 1	Tiputini	1
		sp. 2	Onkone Gare	1
		sp. 3	Onkone Gare	1
		sp. 4	Onkone Gare	1
	* Cnesinus *	sp. 1	Onkone Gare	4
		sp. 2	Onkone Gare	6
		sp. 3	Tiputini	1
		sp. 4	Tiputini	2
		sp. 5	Tiputini	2
		sp. 6	Onkone Gare/Tiputini	4
		sp. 7	Onkone Gare/Tiputini	4
		sp. 8	Onkone Gare	1
		sp. 9	Onkone Gare/Tiputini	5
		sp. 10	Onkone Gare	1
	* Eupagiocerus *	sp. 1	Onkone Gare/Tiputini	2
	* Pagiocerus *	sp. 1`	Onkone Gare/Tiputini	4
	* Sternobothrus *	sp. 1	Tiputini	2
		sp. 2	Onkone Gare	1
		sp. 3	Onkone Gare	1
		sp. 4	Onkone Gare	1
Phloeosinini	* Chramesus *	sp. 1	Onkone Gare	1
		sp. 2	Tiputini	1
		sp. 3	Onkone Gare	1
		sp. 4	Tiputini	1
		sp. 5	Onkone Gare	1
		sp. 6	Onkone Gare	1
Phloeotribini	* Phloeotribus *	sp. 1	Onkone Gare/Tiputini	31
		sp. 2	Onkone Gare	20
		sp. 3	Onkone Gare/Tiputini	6
		sp. 4	Onkone Gare/Tiputini	21
		sp. 5	Onkone Gare	1
		sp. 6	Onkone Gare	4
		sp. 7	Onkone Gare	1
		sp. 8	Onkone Gare	1
		sp. 9	Onkone Gare	2
		sp. 10	Onkone Gare	1
		sp. 11	Onkone Gare	1
		sp. 12	Tiputini	2
		sp. 13	Onkone Gare	1
		sp. 14	Tiputini	1
		sp. 15	Onkone Gare/Tiputini	7
		sp. 16	Onkone Gare	13
		sp. 17	Onkone Gare	1
		sp. 18	Tiputini	1
		sp. 19	Onkone Gare	1
		sp. 20	Onkone Gare	1
		sp. 21	Onkone Gare	1
Phrixosomini	* Phrixosoma *	sp. 1	Onkone Gare	1
		sp. 2	Onkone Gare	1
		sp. 3	Onkone Gare	1
				183

**Table 3.** Numbers of ambrosia beetle species collected at two sites in Ecuador.

Subtribe	Genus	Species	Locality(ies)	No. Specimens
Xyleborini	* Ambrosiodmus *	sp.	Onkone Gare	1
	* Callibora *	*sarahsmithae*	Onkone Gare	1
	* Coptoborus *	sp. 1	Onkone Gare/Tiputini	6
		sp. 2	Onkone Gare	1
		sp. 3	Onkone Gare	2
		sp. 4	Onkone Gare/Tiputini	15
		sp. 5	Onkone Gare/Tiputini	11
		sp. 6	Onkone Gare/Tiputini	2
		sp. 7	Tiputini	1
		sp. 8	Onkone Gare/Tiputini	2
		sp. 9	Onkone Gare/Tiputini	5
		sp. 10	Onkone Gare/Tiputini	6
		sp. 11	Onkone Gare	1
		sp. 12	Onkone Gare	1
		sp. 13	Onkone Gare	1
		sp. 14	Onkone Gare	1
		sp. 15	Onkone Gare	1
		sp. 16	Onkone Gare	1
		sp. 17	Onkone Gare	3
		sp. 18	Onkone Gare	1
		sp. 19	Onkone Gare	1
		sp. 20	Onkone Gare	1
		sp. 21	Onkone Gare	1
		sp. 22	Tiputini	1
		sp. 23	Onkone Gare	1
		sp. 24	Onkone Gare	1
		sp. 25	Onkone Gare	2
		*vespatorius*	Onkone Gare	1
	* Dryocoetoides *	sp. 1	Onkone Gare	6
		sp. 2	Onkone Gare	1
		sp. 3	Onkone Gare	1
		sp. 4	Onkone Gare	1
		sp. 5	Onkone Gare	2
	* Theoborus *	sp. 1	Tiputini	1
		sp. 2	Tiputini	3
		sp. 3	Onkone Gare/Tiputini	5
		nr. micarius	Onkone Gare	1
		sp. 4	Onkone Gare	3
		sp. 5	Tiputini	1
		sp. 6	Onkone Gare/Tiputini	8
		sp. 7	Tiputini	1
		sp. 8	Onkone Gare	1
		sp. 9	Onkone Gare	1
		sp. 10	Onkone Gare	1
	* Xyleborinus *	sp. 1	Tiputini	2
	* Xyleborus *	sp. 1	Tiputini	21
		*spathipennis*	Onkone Gare/Tiputini	2
		*affinis*	Onkone Gare/Tiputini	317
		sp. 2	Tiputini	1
		nr. ferrugineus	Onkone Gare/Tiputini	12
		sp. 3	Onkone Gare	2
Xyleborini	* Xyleborus *	sp. 4	Tiputini	8
		sp. 5	Tiputini	1
		sp. 6	Onkone Gare	1
		sp. 7	Tiputini	1
		sp. 8	Tiputini	1
		sp. 9	Onkone Gare	1
		sp. 10	Onkone Gare	2
		sp. 11	Tiputini	1
		sp. 12	Onkone Gare	1
		sp. 13	Onkone Gare	1
		sp. 14	Onkone Gare	2
		sp. 15	Onkone Gare	1
		sp. 16	Onkone Gare	1
		sp. 17	Onkone Gare	1
		sp. 18	Onkone Gare	1
		sp. 19	Onkone Gare	1
	* Xylosandrus *	*morigerus*	Onkone Gare/Tiputini	12
Premnobiina	* Premnobius *	*cavipennis*	Onkone Gare	1
Total				504

**Figure 1.**
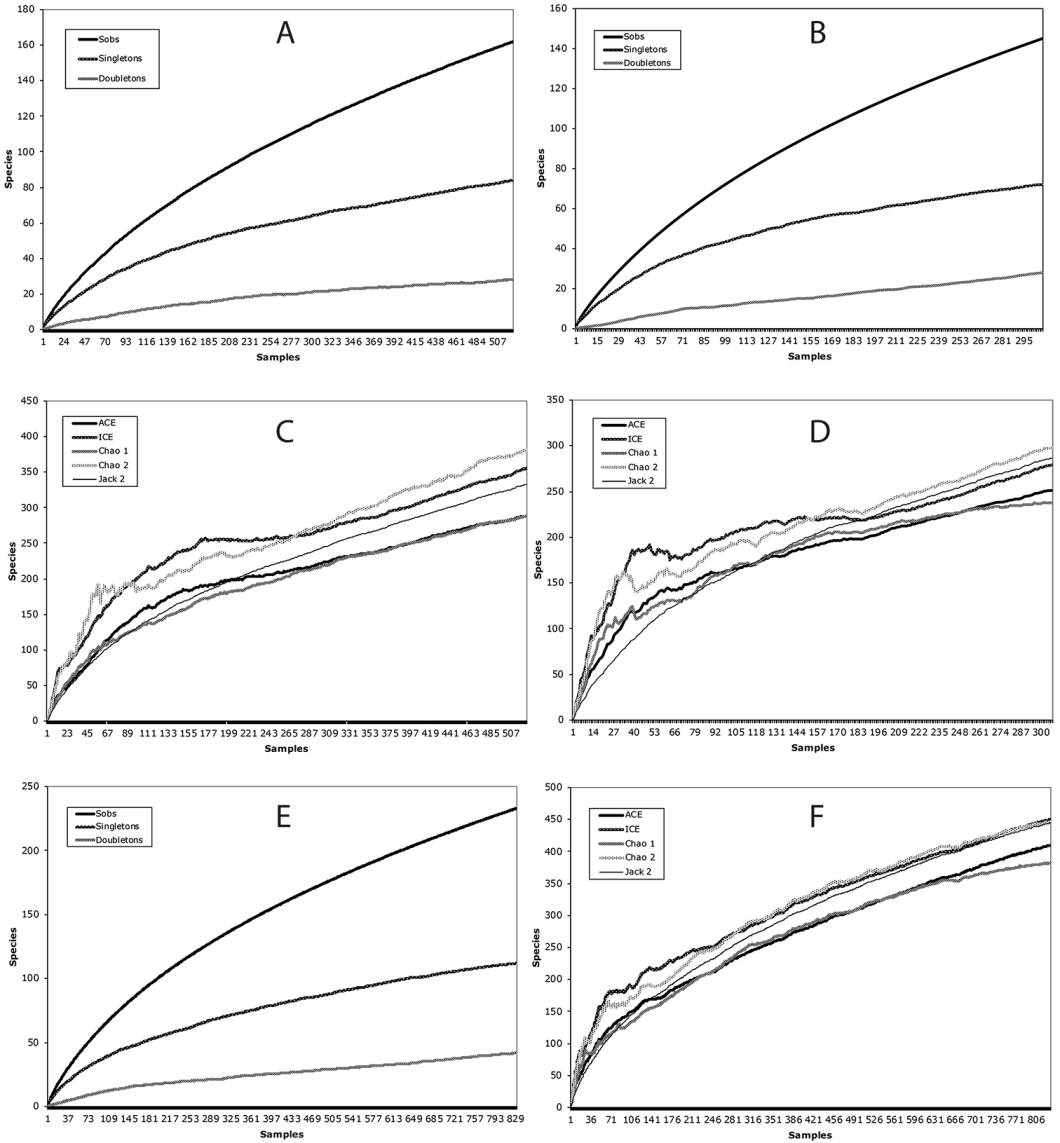
**A** species accumulation curves for Onkone Gare **B** species accumulation curves for Tiputini **C** species richness estimators for Onkone Gare **D** species richness estimators for Tiputini **E** species accumulation curves for both sites combined **F** species richness estimators for both sites combined.

Species accumulation curves for both sites combined and for each site individually did not reach an asymptote (Fig. 1). The steady increase of the curves indicated a continued accumulation of rare species (singletons and doubletons). Estimates of species richness were very similar between abundance-based and incidence-based statistics (Table 1; Fig. 1). For the two sites combined, the abundance-based ACE and Chao1 estimated a total species richness of 301 and 308 species, respectively. The incidence-based ICE and Chao2 gave marginally higher estimates. Abundance-based and incidence-based estimates of species richness for the Onkone Gare overlapped slightly with 275–309 species. Abundance-based and incidence-based (Chao1) statistics arrived at similar estimates and the incidence-based statistics, ICE and Chao2 estimated a slightly greater species diversity for the Tiputini site. The second-order Jackknife estimated a species richness of 260 species for both study sites (Table 1; Fig. 1). For Onkone Gare, the second-order Jackknife estimated 217 species and for Tiputini it estimated 95 species.

Simple Complementarity Indices suggested that the composition of the scolytine fauna was markedly different for the two study sites. Onkone Gare and Tiputini had a CI = 0.81 for the total analyzed subset of scolytine tribes (Table 1). Complementarity Indices for the tribes ranged from 0.73 to 1.00 (although a CI=1 for Premnobiina is meaningless because only one specimen was collected) (Table 5). In the samples, at least half of all the species occurring at either site was unique to that site: 76% of species at Onkone Gare and 50% or species at Tiputini (Table 1).

However, the correction for biases of incomplete sampling of the fauna using the Chao-Sørensen abundance-based estimator with the rarefied data estimated a faunal similarity of 0.79. Similarly, the Monte Carlo analysis returned a probability of 0.374 [median modeled similarity L’ = 0.77 (lower and upper 2.5% quantiles = 0.634 and 0.836)]. Both the Chao-Sørensen and the Monte Carlo analyses indicate that the apparent difference between the two sites was due to stochastic sampling error and the difference does not appear to be biologically significant.

**Table 4.** South American (SA) scolytine genera and species recorded and collected via canopy fogging.

Taxon	SA Genera	SA Species	Genera Collected	Species Collected
Xyleborini	11	233	8	69
Premnobiina	2	4	1	1
Bothrosternini	6	82	6	22
Phloeosinini	5	61	1	6
Phloeotribini	1	54	1	21
Phrixosomini	1	10	1	3

**Table 5.** Distribution of species by taxon between sites and the corresponding index values.

Taxon	% Occurrence in Samples	Onkone Gare spp.	Tiputini spp.	Total spp.	Shared spp.	Unique spp.	CI
Xyleborini	27	56	27	69	14	55	0.81
Premnobiina	0.09	1	0	1	0	1	1.00
Bothrosternini	4.32	17	11	22	6	16	0.73
Phloeosinini	0.52	4	2	6	0	6	1.00
Phloeotribini	7.69	18	7	21	4	17	0.81
Phrixosomini	0.26	3	0	3	0	3	1.00

## Discussion

The goals of this study were to assess the scolytine species richness, the use of canopy fogging to enhance the discovery of new taxa, and to estimate the faunal turnover (β-diversity) between a short distance in Amazonian rain forest. Species records for Ecuador range from 50 recorded in a monograph (Wood 2007) to 85 collecting at one site over a year (Martinez et al. 2019) and 248 have been recorded for Peru (Smith et al. 2017). Our entire collection of scolytines from the fogging represents 400 morphospecies out of 2500 specimens. This species richness is proportionally 30x greater than the species richness recorded for a western Ecuadorian forest (85 species out of ~18,000 specimens). Given the estimates of species richness suggest nearly twice the number of species in only these field sites (Tables 2, 3) and the diversity and long stability of Ecuadorian forest habitats (McKenna and Farrell 2006), it is likely the actual scolytine diversity exceeds 400 species.

Taxonomic study of these 400 morphospecies has yielded species descriptions of 21 *Scolytodes* species (84% of the known Ecuadorian fauna) (Jordal and Smith 2020), seven *Camptocerus* species (39% of the known Ecuadorian fauna) (Smith and Cognato 2010), and 15 *Coptoborus* (45% of the known Ecuadorian fauna) (Smith and Cognato, in prep.). In addition, faunal discoveries include descriptions of new genera with striking morphologies: *Akrobothrus* Dole & Cognato, 2007 and *Callibora* Cognato, 2018. These discoveries suggest that canopy fogging is not only a useful method for the collection of known scolytine fauna, but it is also a mean to access a poorly explored fauna.

Our analysis of the partial scolytine samples fogged from the Ecuadorian Amazonian canopy indicates a species-rich fauna but the estimate of a high β-diversity depended on the method’s sensitivity to undersampling. Specifically, the simple measure of complementarity suggested substantial faunal difference between the sites, but the Chao-Sørensen and Monte Carlo resampling showed that the differences were statistically inconclusive.

Our results indicate that even a large-scale, long-term sampling effort, such as this one, did not provide a reliable estimate of scolytine diversity for the two western Amazonian sites. Despite over 1100 individual fogging events (representing 14 fogging expeditions), which collected 688 individual beetles representing 121 species (used in this analysis), the species accumulation curves did not reach an asymptote (Fig. 1). Likewise, the accumulation of rare species in the form of singletons and doubletons did not decline. These phenomena were observed from the individual data collected for each study transect and for the collective species data for the two sites.

Despite this inconclusive result, we speculate on the scolytine diversity in the canopy. Given the relatively short distance (21 km) between Onkone Gare and Tiputini the real β-diversity is likely low because of the sites’ relatively proximity and very similar environment. The Onkone Gare and Tiputini sites differ in the occurrence of tree species but share the same tree families (Erwin et al. 2005). Bark and ambrosia beetle’s tree host specificity usually occurs at the family level (Beaver 1979) thus predicting low β-diversity between these sites. Conversely, previous canopy fogging studies have found significant differences in the beetle species composition of different forest types. In Manaus, Brazil, 83% of beetle species in canopy fogging samples were found in only one type of forest (Erwin 1983a). Of the four forest types characterized and studied by Erwin, “mixed-water” forests were found to be the most species rich. However, “terra firme” (non-floodplain) forests had the second highest number of species and the highest number of restricted species. Both sites sampled in this study were “terra firme” forest. Erwin also found that smaller insect species (1 mm class, as defined for his study) comprise the majority of insects in “terra firme” forests. This offers another predictor of scolytine diversity in this habitat, as most scolytine species are within the 1–3 mm range. If scolytines follow the same general patterns found for all Amazonian insects, we can predict that further sampling would uncover an even richer scolytine fauna with possibly many species not found in other forest types. The high percentage of singletons and doubletons at each site supports this prediction. Conversely, increased sampling may yield more specimens of rare species which would decrease β-diversity.

Large-scale sampling of tropical habitats offers a rich source of previously unknown species, but as this study demonstrates, even the most ambitious sampling schemes may not be enough to adequately assess the true species richness of hyper-diverse groups. Here we have uncovered a level of scolytine α-diversity that increases the known fauna of Ecuador nearly five-fold, only based on sampling a single Amazonian habitat. Future studies will need even more extensive sampling protocols to avoid erroneous conclusions and over-estimates of β-diversity based on stochastic sampling (Hulcr et al. 2007; Lewinsohn and Roslin 2008).
